# Eosinophilic Granulomatosis With Polyangiitis Presenting as Bilateral Orbital Inflammation

**DOI:** 10.7759/cureus.83131

**Published:** 2025-04-28

**Authors:** Lisa Zhu, Tamara Dahhan

**Affiliations:** 1 Department of Medicine, University of California Los Angeles (UCLA) Medical Center/David Geffen School of Medicine, Los Angeles, USA

**Keywords:** elevated serum igg4, eosinophilic granulomatosis with polyangiitis (egpa), nucala, orbital swelling, small vessel vasculitis

## Abstract

Eosinophilic granulomatosis with polyangiitis (EGPA) is a small vessel vasculitis that most commonly presents with peripheral eosinophilia, upper airway disease, and asthma, and can also cause cutaneous, cardiac, neurologic, musculoskeletal, gastrointestinal, and renal disease. This case describes a 57-year-old male with a history of chronic sinusitis and asthma who presented with bilateral orbital inflammation causing proptosis and diplopia. He was initially treated with antibiotics for orbital cellulitis without benefit, then high-dose prednisone with taper for idiopathic orbital inflammation, with resolution of ocular symptoms. He was diagnosed with EGPA after rheumatologic evaluation. Serum Immunoglobulin G4 (IgG4) level was significantly elevated, but his clinical presentation was more consistent with EGPA than IgG4-related disease (IgG4-RD). The patient significantly improved with the addition of mepolizumab and later methotrexate. This case details a rare manifestation of EGPA-bilateral orbital inflammation and highlights the potential overlapping features between EGPA and IgG4-RD.

## Introduction

Eosinophilic granulomatosis with polyangiitis (EGPA) is a rare autoimmune disease within the family of anti-neutrophil cytoplasmic antibody (ANCA)-associated vasculitides. EGPA causes multi-system inflammation, typically progressing through allergic, eosinophilic, and then vasculitic stages [1]. ANCAs cause microvascular inflammation by activating neutrophils, promoting the development of neutrophil extracellular traps, and activating the alternative complement pathway [2]. Ophthalmic involvement is relatively rare in EGPA, with an estimated prevalence of 5-20% [2] compared to 50% in granulomatosis with polyangiitis (GPA) [3]. However, EGPA can cause serious ophthalmic manifestations and should be considered in the differential diagnosis for orbital inflammation/vasculitis. IgG4-related disease (IgG4-RD) is another systemic autoimmune disease that can have orbital and periorbital involvement. Accurately distinguishing between the two is important as their treatment pathways differ. Serum Immunoglobulin G44 (IgG4) levels are often elevated in active EGPA [4]; this observation helped provide clarity in this case of orbital inflammation with EGPA features and elevated serum IgG4.

## Case presentation

A 57-year-old male with a past medical history notable for chronic sinusitis and asthma presented to the rheumatology clinic with bilateral orbital inflammation. In his thirties, he developed asthma, chronic sinusitis, and nasal polyposis requiring multiple sinus surgeries. He had several years of joint pain with stiffness but no swelling, affecting the shoulders, hips, knees, and ankles. Five months prior to presentation, he was diagnosed with a sinus infection, which persisted despite multiple courses of antibiotics. Two months prior to presentation, he developed progressive bilateral periorbital swelling and erythema, with right-sided proptosis and intermittent diplopia, low-grade fevers, and night sweats. He was seen by his primary care doctor and ophthalmologist and was treated with doxycycline and cetirizine without improvement, as well as multiple methylprednisolone dose packs with transient improvement.

Laboratory studies (Table 1) performed upon presentation to the rheumatology clinic were notable for elevated sedimentation rate (54 mm/Hr) and C-reactive protein (6.5 mg/dL), leukocytosis (white blood cell count 20.1 K/uL) with peripheral eosinophilia (absolute eosinophil count 8.2 K/uL; note he had mild intermittent eosinophilia for five years prior to presentation), positive cytoplasmic anti-neutrophil cytoplasmic antibody (C-ANCA) 1:40 and myeloperoxidase (MPO) antibody (472 CU), elevated serum IgG (1795 mg/dL) and serum IgG4 (631 mg/dL). Perinuclear anti-neutrophil cytoplasmic antibody (P-ANCA), proteinase-3 (PR3) antibody, antinuclear antibody, thyroid-stimulating hormone, and angiotensin-converting enzyme were normal, as were serologies for Strongyloides and other parasites.

**Table 1:** Key laboratory abnormalities

Test	Result	Reference range	Interpretation
White blood cell count	20.1 K/uL	4.5-11 K/uL	Leukocytosis
Absolute eosinophil count	8.2 K/uL	≤0.5 K/uL	Severe eosinophilia
Erythrocyte sedimentation rate	54 mm/Hr	≤12 mm/Hr	Elevated
C-reactive protein	6.5 mg/dL	<0.8 mg/dL	Elevated
C-ANCA (cytoplasmic anti-neutrophil cytoplasmic antibody)	1:40	<1:20	Positive
Myeloperoxidase antibody	472 CU	<20 CU	Positive
Serum immunoglobulin G (IgG)	1795 mg/dL	700-1600 mg/dL	Elevated
Serum immunoglobulin G4 (IgG4)	631 mg/dL	1-123 mg/dL	Elevated

**Figure 1:**
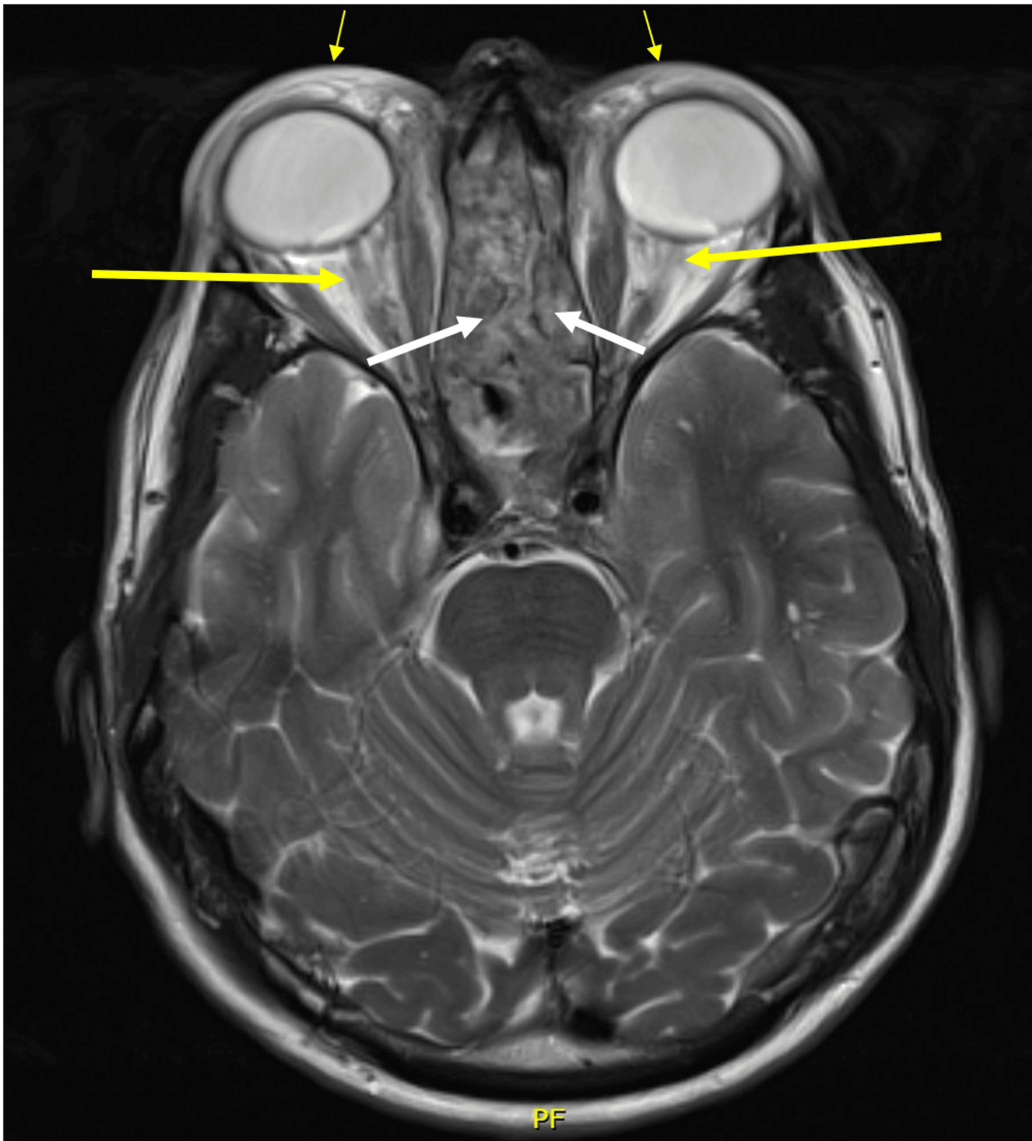
Axial MRI of the orbits MRI (axial view) of the orbits showed bilateral preseptal edema (thin yellow arrows), orbital edema (thick yellow arrows), and pan-sinusitis (white arrows).

Magnetic resonance imaging (MRI) of the brain and orbits showed severe edema and enhancement throughout both orbits and profound sinonasal cavity mucosal thickening (Figure 1). He underwent sinus biopsy that showed chronic rhinosinusitis with severe eosinophilic infiltration and focal areas of small vessel eosinophilic vasculitis, without necrosis or granulomatous inflammation. His ophthalmologist started him on prednisone 60 mg daily for idiopathic orbital inflammatory syndrome.

Upon presentation to the rheumatology clinic, the patient had experienced near resolution of ocular symptoms and other presenting symptoms. Vitals were within normal, and physical exam was notable only for minimal swelling of the right upper eyelid. He was diagnosed with EGPA based on peripheral eosinophilia, positive C-ANCA and MPO, elevated inflammatory markers, chronic sinusitis, nasal polyposis, asthma, bilateral orbital inflammation, and polyarthralgia. His five-factor score (a tool used to assess prognosis in patients with vasculitis, where a score ≥1 is associated with significantly higher mortality) was zero [5].

Orbital cellulitis, Graves’ ophthalmopathy, lymphoma and other malignancies, vasculitis, IgG4-RD, sarcoidosis, Tolosa-Hunt syndrome, and idiopathic orbital inflammation were considered in the differential for orbital inflammation [6]. Many of these conditions were excluded based on his laboratory results. IgG4-RD or overlap was considered; however, he lacked other IgG4-RD features and had already been on high-dose prednisone for three weeks; as a result, orbital biopsy was not pursued. He underwent a bone marrow biopsy that was negative for hematologic malignancy, and his hematologist did not feel that a Positron Emission Tomography-Computed Tomography (PET-CT) scan was necessary.

Prednisone was gradually tapered with the plan to add mepolizumab, an interleukin-5 antagonist, pending evaluation for cardiomyopathy. As prednisone was tapered to 20 mg daily, he experienced recurrent periorbital inflammation, sinus congestion, and joint pain that were milder than his initial presentation. Therefore, mepolizumab 300 mg subcutaneous every four weeks was added. After six months of mepolizumab, the patient had normalization of his peripheral eosinophilia, C-ANCA, MPO, and inflammatory markers, and was able to taper off prednisone without recurrent orbital inflammation. Despite his significant improvement, his asthma, sinusitis, and arthralgia were still active, and methotrexate was added with further improvement. He continues to follow in the rheumatology clinic with good disease control, without development of any other clinical or radiologic features of IgG4-RD.

## Discussion

EGPA is a small vessel vasculitis within the family of ANCA-associated vasculitis. It is a rare disease with an estimated prevalence of 10-14 per million people and a mean age of onset at 50 years, characterized by eosinophilia with multi-system inflammatory involvement [1]. The vast majority of patients with EGPA have asthma, usually adult-onset. Other potential manifestations include chronic rhinosinusitis, nasal polyposis, pneumonia, diffuse alveolar hemorrhage, palpable purpura, peripheral neuropathy, cardiomyopathy, glomerulonephritis, gastroenteritis, and arthritis. The disease classically progresses in three stages - allergic, eosinophilic, and vasculitic; though there may be overlap between phases [2]. The allergic phase is characterized by allergic rhinitis, asthma, and atopic disease. The eosinophilic phase is characterized by blood and tissue eosinophilia, for example, in the lungs, heart, and gastrointestinal tract. Systemic small- and medium-vessel vasculitis can also present later, in the vasculitic phase [1].

Because vasculitis does not present until later in the disease course, diagnosis of EGPA can be delayed. Unlike in the other ANCA vasculitides, ANCA serologies are positive in only 30-40% of patients with EGPA [1]. ANCA-positive patients are more likely to have vasculitic manifestations, whereas ANCA-negative patients are more likely to have prominent eosinophilic manifestations like cardiomyopathy. All patients with newly diagnosed EGPA should get an echocardiogram to screen for cardiac involvement, as this is the major cause of mortality. The differential diagnosis for EGPA includes hypereosinophilic syndromes, allergic bronchopulmonary aspergillosis, chronic eosinophilic pneumonia, other vasculitides, infections, and malignancies.

Ophthalmic involvement in EGPA, while rare, can be severe and vision-threatening. Manifestations can be categorized as idiopathic orbital inflammation-like or ischemic vasculitis. Patients with the former can present with periorbital edema and/or diplopia, whereas patients with the latter can present with retinal artery occlusion, retinal vein occlusion, optic neuritis, and retinal vasculitis [7].

In this case of bilateral orbital inflammation, which is more commonly seen with IgG4-RD than EGPA, there was some diagnostic uncertainty given the patient’s elevated serum IgG4. Cases involving an overlap of ANCA vasculitis and IgG4-RD have been reported [8]. EGPA and IgG4-RD do share some features, such as history of allergic disease, elevated serum Immunoglobulin E (IgE), and elevated serum IgG4, but the pathogenesis of each disease differs substantially [9]. The patient did not meet IgG4-RD classification criteria [10] given the lack of clinical criteria, whereas he did meet EGPA classification criteria [11] based on small vessel eosinophilic vasculitis on sinus biopsy, asthma, nasal polyposis, and peripheral eosinophilia. Elevated serum IgG4 is non-specific and not diagnostic on its own, and can be seen with other inflammatory/autoimmune diseases, malignancy, and infection. Moreover, serum IgG4 is frequently elevated in active EGPA and can be considered a marker of EGPA disease activity. It is postulated that this is due to the role of the T helper 2 (Th2) response in EGPA and the production of IgG4 antibodies [4].

Regarding the patient’s treatment course, mepolizumab was chosen as the first-line steroid-sparing agent based on the MIRRA trial [12]. Induction therapy for EGPA depends on the severity of the disease. Severe/life-threatening organ manifestations are treated with high-dose corticosteroids plus rituximab or cyclophosphamide. Non-severe disease can be treated with corticosteroids plus mepolizumab, methotrexate, azathioprine, or mycophenolate. Mepolizumab is the preferred treatment for non-severe EGPA and acts by impairing eosinophil maturation and survival [13]. The initial goal is to achieve disease remission, then immunosuppressive therapy is continued to maintain remission.

## Conclusions

EGPA should be considered in a patient with peripheral eosinophilia and asthma who develops new manifestations such as palpable purpura, pulmonary infiltrates, peripheral neuropathy, cardiomyopathy, glomerulonephritis, or, less commonly, orbital inflammation, as in this case. Among the ANCA-associated vasculitides, orbital inflammation is more common in GPA, but can rarely occur in EGPA. Prompt referral to a rheumatologist should be considered in a patient with orbital inflammatory disease, especially when their history is suggestive of an underlying systemic process, with the goal of earlier diagnosis and treatment. IgG4-RD can also cause orbital and periorbital inflammation, so an elevated serum IgG4 level (which is common in both IgG4-RD and EGPA) needs to be taken in the context of the other disease manifestations as well as histologic findings on biopsy, if feasible. In a patient with EGPA, an isolated serum IgG4 without other clinical or histologic features of IgG4-RD may be attributed to EGPA disease activity.
